# Impact of genetic mutations and nutritional status on the survival of patients with colorectal cancer

**DOI:** 10.1186/s12885-019-5837-4

**Published:** 2019-06-29

**Authors:** Mariana Abe Vicente Cavagnari, Tiago Donizetti Silva, Marco Antonio Haddad Pereira, Luísa Jacques Sauer, David Shigueoka, Sarhan Sydney Saad, Katia Barão, Carla Caroline Dias Ribeiro, Nora Manoukian Forones

**Affiliations:** 1 Department of Medicine. Gastroenterology Division. Oncology Group, Universidade Federal de São Paulo - SP. Brazil, R Botucatu 740, 2° andar CEP 04023900, Vila Clementino, São Paulo, Brazil; 2 Department of Radiology, Universidade Federal de São Paulo, São Paulo, Brazil; 3 Department of Surgery, Universidade Federal de São Paulo, São Paulo, Brazil

**Keywords:** Sarcopenia, Visceral adipose tissue, Phase angle, Body composition, Oncogene, Tumor supressor gene

## Abstract

**Background:**

The prognosis of colorectal cancer (CRC) patients can be influenced by genetic mutations and nutritional status. The relationship between these variables is unclear. The objective of the study was to verify the variables involved in the nutritional status and genetic mutations, which correlate with survival of CRC patients.

**Methods:**

Patients with surgical intervention for tumor resection were evaluated using body mass index, nutritional screening, patient self-produced global subjective assessment, phase angle, and computed tomography to calculate the areas of visceral adipose tissue (VAT) and subcutaneous adipose tissue, and muscle mass for the determination of sarcopenia. Ten gene mutations involved in CRC carcinogenesis were studied (*PIK3CA, KRAS, BRAF, EGFR, NRAS, TP53, APC, PTEN, SMAD4*, and *FBXW7*). DNA was extracted from fresh tumor or paraffin tissues.

**Results:**

Of the 46 patients, 29 (64.4%) were at nutritional risk and 21 (45.7%) were moderately malnourished. However, there was a high percentage of VAT in 24 (61.5%) and sarcopenia in 19 (48.7%) patients. These variables were associated with a higher risk of mortality. Nutritional risk, moderate or severe malnutrition, phase angle < 5°, VAT < 163.8 cm^2^ in men and <  80.1 cm^2^ in women, and sarcopenia were associated with the relative risk of death, with respective hazard ratios/odds ratios and 95% confidence intervals of 8.77 (1.14–67.1), 3.95 (1.11–14.0), 3.79 (1.10–13.1), 3.43 (1.03–11.4), and 3.95 (1.06–14.6). Increased VAT was associated with a lower risk of death, even in patients older than 60 years or those harboring mutated *KRAS*.

**Conclusions:**

Patients with positive indicators for malnutrition or risk of malnutrition had an increased risk of death. No relationship was identified between the presence of mutations and survival.

## Background

Genetic alterations that have been described in colorectal cancer (CRC) include chromosomal instability (CIN), which generates sporadic CRC, microsatellite instability (MSI) represented by known hereditary syndromes [1, 2], and a third serrated pathway of CpG island methylation (CIMP). [3] High body mass index (BMI) is another established risk factor for CRC, [1, 4] but the impact of BMI is unclear, possibly due to the timing of the BMI assessment in relation to the diagnosis. [5, 6] Emerging data suggests that the association of BMI with CRC differs by MSI status of the tumor. [1, 2] Therefore, higher BMI is commonly associated with a lower MSI frequency. Thus, microsatellite stability (MS) is frequently observed in obese individuals with CRC, with consequent reduction in the presence of mutations. This condition may be related to several factors, including the high levels of cytokines that accompany obesity. [4] MSI is an established marker of survival for patients with CRC. Subjects with high MSI tumors have a favorable prognosis in comparison with age and patients at matched stage with stable tumors in MS. [3, 7] Similarly, patients with mutated *KRAS* and *BRAF* tumors have a worse prognosis compared to patients harboring wild type *KRAS* and *BRAF*. [3, 8]

The impact of BMI on the survival of patients with CRC is controversial. Increased BMI has been associated by some authors with short survival in some cancers, such as CRC. [9] In contrast, other studies have reported lower mortality among overweight or moderately obese patients with CRC. [10, 11] However, the combination of several methods, such as the use of BMI, bioelectrical impedance analysis, computed tomography for the analysis of sarcopenia and visceral fat, Nutritional Risk Screening (NRS), and the subjective Patient-Produced Subjective Nutritional Assessment (PG-SGA), have demonstrated more conclusive responses in predicting survival in patients with CRC. [5, 10, 11]

These contradictory findings suggest a potential obesity paradox, preventing a conclusive interpretation of its role in predicting overall cancer survival. Molecular changes, such as the presence of mutations, present clearer evidence regarding the prognosis of colon and rectum adenocarcinoma. However, consistent evidence suggests that the nutritional status in peri- and post-diagnosis periods of these individuals also influences the prognosis related to the disease. [12] The available evidence indicates the importance of genetic information associated with the nutritional status of patients with CRC, although there are few related studies as well as studies of gene and body composition variables. [13–15] Morikawa et al. [14] verified the association of *TP53* gene and BMI in patients with CRC. However, the authors did not find a significant correlation of mutated *TP53* and survival. However, in non-obese individuals (BMI < 30 kg/m^2^), the presence of a mutation in *TP53* has been associated with poor survival, with no significant association with survival in patients with BMI > 30 kg/m^2^. Thus, the authors concluded that the survival of CRC patients with mutations in *TP53* differs significantly according to their BMI. [14] This occurs because the tumor suppressor gene is induced in a cellular response to the reduction of nutrients or energy levels, thus avoiding cell proliferation under nutrient deprivation conditions. [16]

Epidemiological studies suggest that the causal effects of obesity or excess energy balance are associated with the incidence of colon cancer and mortality. [4, 5] It has been suggested that *TP53* associated with the energy balance influences tumor behavior in a manner that is optimized in relation to the absence of mutations. [14] The same study also verified the presence of mutations in other genes, although it was limited only to gene examinations.

**Fig. 1:**
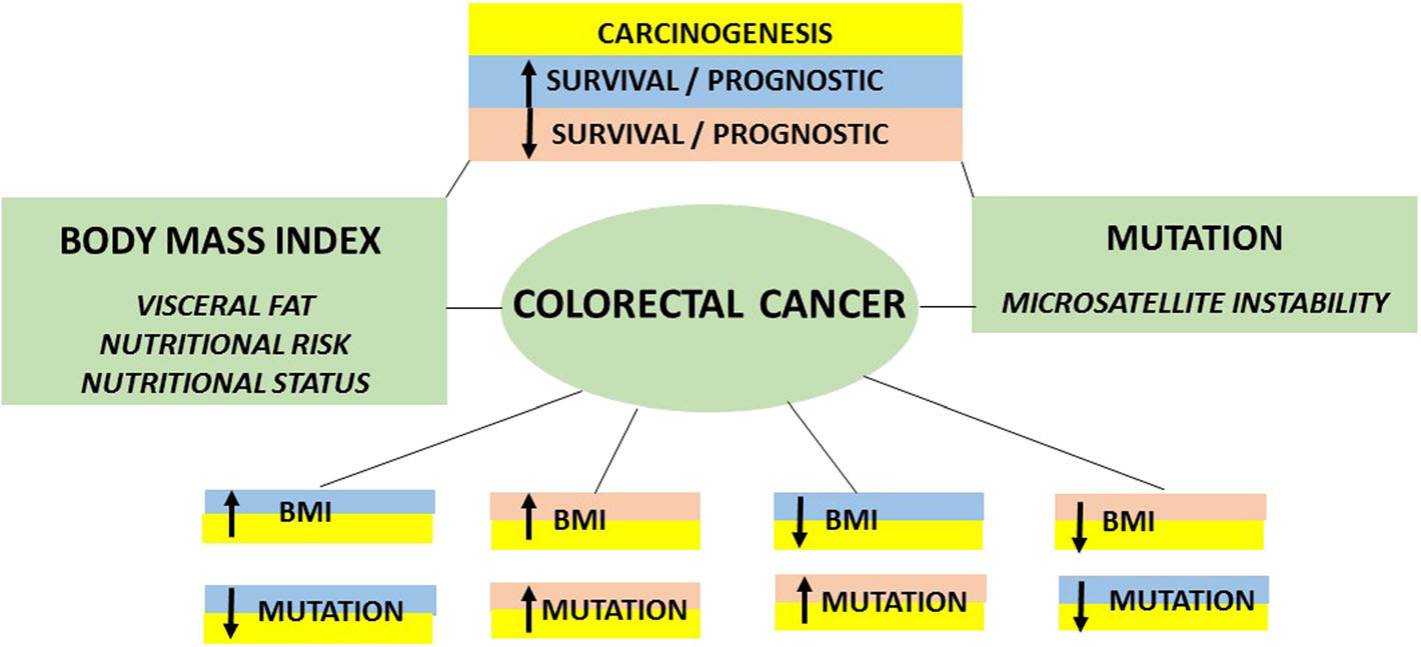
The carcinogenesis, survival and prognostic of colorectal cancer patients can be influenced by genetic mutations and nutritional status Those process involves interactions among increased BMI and low mutations, increased BMI and high mutations, reduced BMI and high mutations or reduced BMI and low mutations

We have included genes that may interfere in the carcinogenesis and prognosis of CRC (*BRAF, KRAS, NRAS, EGRF, PIK3CA, PTEN, APC, SMAD4,* and *FBXW7*). The data indicated that the prognosis of CRC patients can be influenced by genetic mutations and nutritional status (Fig. 1). To date, however, the relationship between these variables has been poorly documented. The objective of the present study was to verify the variables involved in the nutritional status and genetic mutations, which correlate with the survival of patients with CRC.

## Methods

### Study participants

This cross-sectional study was based considering the number of patients eligible for CRC surgery from July 2013 to April 2016 at Hospital Sao Paulo. Patients with a diagnosis of CRC with a surgical intervention proposal for cancer resection were evaluated for the study. The time between CRC diagnosis and surgery ranged from 20 days to 18 months. The study was approved by the local Ethics Committee (Protocol 304.754/2013).

### Data collection

The sex, age, treatment, site, death and tumor stage data were obtained from the patient medical records.

At the preoperative time the patients were evaluated in a single moment ranged 20–60 days before surgery by means of nutritional variables in order to analyze the nutritional status and the body composition. Tumor samples of 1cm^2^ were collected at the surgical center under the responsibility of a single pathologist. In the impossibility of collecting fresh tumor tissue, paraffin tissues were analyzed.

### Assessment of nutritional status

The BMI was calculated as weight (kg) divided by height (m^2^) and classified according to the World Health Organization criteria [17]. Patients older than 60 years were classified according to BMI for the elderly according to Pan American Health Organization criteria [18].

The NRS 2002 is used to predict outcome based on risk parameters identified in the nutritional assessment. Patients are classified as at nutritional risk, when they obtain a sum of > 3 points. [19]

The validated Portuguese version of the scored PG-SGA was used to assess nutritional status. Subjective analysis classified the patients into three categories: (A) well-nourished, (B) moderately undernourished or suspected of being undernourished, and (C) severely undernourished. [20]

The phase angle (PA) was calculated as the ratio between resistance (R) and reactance (Xc) determined with QuadScan 4000 instrument (Bodystat Ltd., Isle of Man) which applies a 200 μA current at frequencies of 5, 50, 100, and 200 kHz. The PA for the whole body at 50 kHz was calculated from the impedance values using software supplied by Bodystat Ltd. All procedures and control for other variables affecting the validity, reproducibility and precision of the measurements were performed according to the National Institutes of Health guidelines. [21]

### Body composition

Body composition was measured with SliceOmatic Software Horos v2.0.1 using routine CT scans conducted for diagnostic purposes before surgery ranged 20–60 days. The study of the images was performed by a single trained radiologist, according to the methodology proposed by Mourtzakis et al. [22]. The studied image was acquired from the abdominal cross section at the level of the third lumbar vertebra (L3), where the transverse processes were better visualized, from which the following areas were calculated: subcutaneous adipose tissue (SAT), visceral adipose tissue (VAT) and skeletal muscle tissue (SMT).

The cross-sectional areas were measured semi-automatically using the “grow region” tool (2D / 3D Segmentation) in the “region of interest” (ROI) menu, based on differences in Hounsfield (UH) units between structures. The intervals used were -190UH to -30UH for the SAT and the VAT and from -30UH to 110UH for the skeletal musculature. When not every area was computed, due to the discontinuity between structures, more than one region of interest was generated, and the subsequent merge was performed using the “select all ROIs in the series” tool and subsequently the “merge melected ROIs” tool menu “ROI” and submenu “BRUSH ROIs”). Possible overlapping areas of the structures, overestimating the areas, were corrected manually with the tool “BRUSH”.

From the calculated areas, SAT and VAT, some classifications were performed. The high VAT classification was determined according to the proposal of Doyle et al. [23] > 163.8 cm^2^ for men and > 80.1 cm^2^ for women.

Through the VAT and SAT, the ratio between these areas was verified and if > 0.4 cm^2^, the diagnosis of visceral obesity for both genders was confirmed [24]. For SAT, no cutoff values have been reported in the literature.

With the skeletal muscle area, it was possible to calculate the skeletal muscle index (SMI), that was calculated as the ratio of skeletal muscle area (cm^2^) / height (m)^2^ to allow it to be classification of sarcopenia. It should be noted that secondary sarcopenia or sarcopenia associated with cancer was evaluated, since objective tests for functional capacity testing will not be applied. [25] Patients were considered sarcopenic according to Martin et al. [26]

### Study of mutations

The TruSight® Tumor 26 is a kit inventoried by Illumina that allows through NGS technology to take a deeper view of the variations in solid tumors. In the essay not only point mutations in hot spots are analyzed, offering a more comprehensive view of the somatic variations. TruSight Tumor 26 provides primers targeting specific regions of interest, so a full gene sequencing was not performed, but rather the search for specific changes, such as: Kras: Exon 2, 3 and 4; BRAF: G455R; Q456R; V600E and V459. For the study the construction of DNA libraries for new generation sequencing (NGS), the TruSight™ Cancer Sequencing Panel was used for analysis of *KRAS, PIK3CA, BRAF, EGFR, NRAS, TP53, PTEN, APC, SMAD4* and *FBXW7* mutations in DNA from 1 cm^2^ of frozen fresh tumor tissue from the surgical fragments and 1 mm formalin-fixed paraffin-embedded tumor tissue taken from areas with 90% tumor cells as previously described.

In brief, genomic DNA was extracted from tumor tissue using QIAamp MinElute spin columns (Qiagen) and DNA regions of interest were PCR amplified (Veriti 96 Well Fast Thermal Cycler, Applied Biosystems Inc., Foster City CA). The variants present in the 10 genes evaluated in this study were manually filtered, named according to ClinVar analyzed one by one as to their type and classified according to their pathogenicity based on the deposits of databases. The variants that were not deposited or classified by the 12 databases, as well as those classified as of uncertain significance -variant of unknown significance (VUS) were evaluated in the predictive programs of effect of variants Sift and PolyPhen.

### Statistical analysis

Statistical analysis was performed using the statistical software SPSS 20.0 (SPSS Inc., Chicago, IL, USA) and Minitab 16. Continuous variables were tested for normality by the Shapiro-Wilk test for subsequent selection of the statistical tests to be used. Continuous variables were compared using Student’s t-test. Survival was defined between the time of CRC diagnosis until the date of death from any cause. The Kaplan-Meier method was used to calculate survival and compared by the Log-Rank test. The variables that presented *p* <  0.20 were selected for the univariate and multivariate Cox Proportional Regression. The resulting variables were expressed as “Hazard Ratio”. The risk indicators were then adjusted according to variables of interest. A level of significance of 5% (*p* <  0.05) and confidence interval of (95%) were used.

## Results

Forty-six CRC patients were included. The mean age of the patients was 62.8 + 13 years and 34 (73.9%) were female. The distribution ratio of tumor sites was similar (*p* = 0.978). Tumors were found in the ascending and transverse colon in 15 (32.6%), descending and sigmoid colon in 16 (34.8%), and rectum in 15 (32.6%) patients. According to the TNM classification, [27] 5 (10.8%), 11 (23.9%), and 12 (26.2%) patients were in stage I, II, and III, respectively, with the majority of patients in stage IV (*n* = 18, 39.1%). It should be emphasized that the purpose of the study was to evaluate patients with an intention of surgical resection of the CRC. Although, many patients received an indication of surgery, the procedure was later contraindicated due to disease progression.

**Fig. 2:**
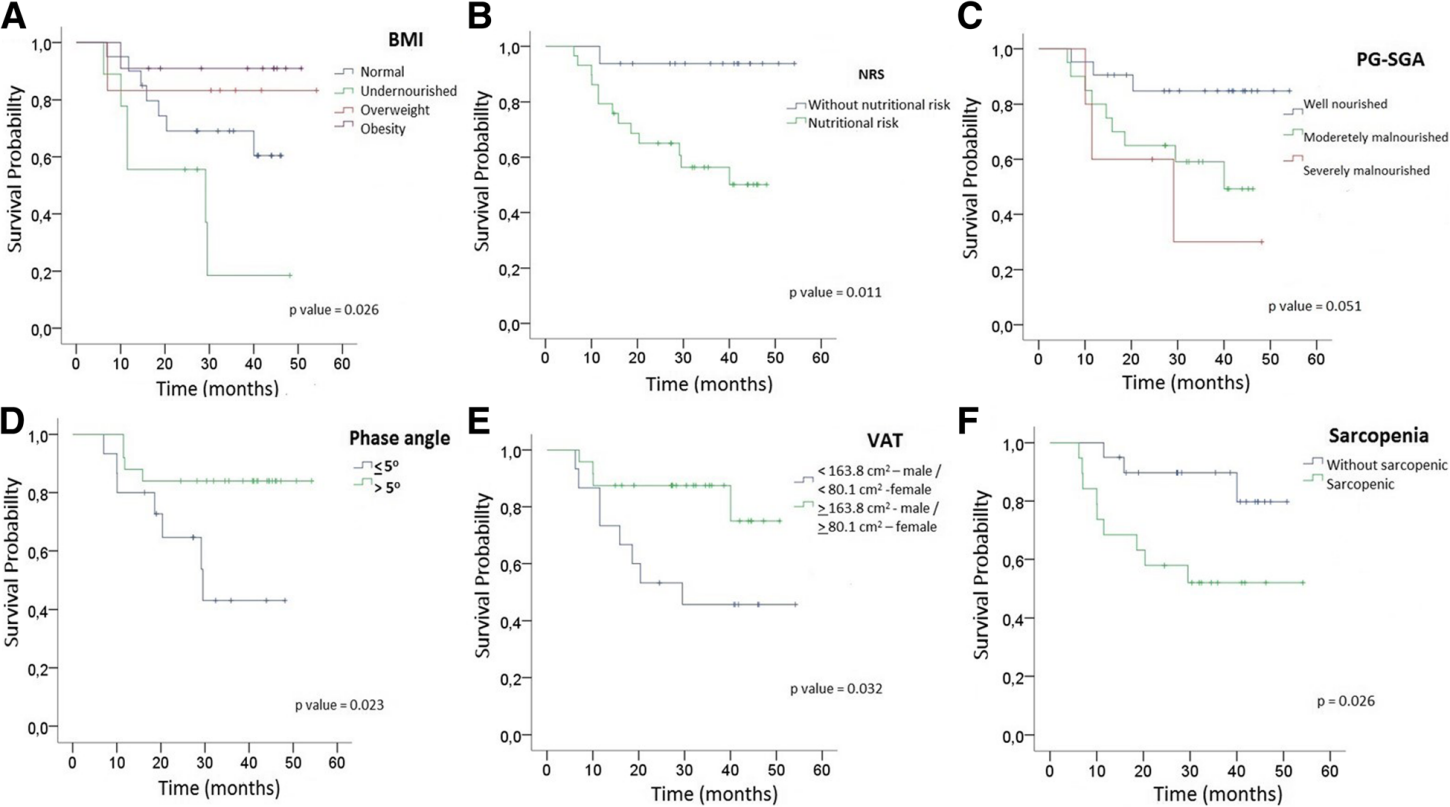
Kaplan Meier curves of nutritional indicators. a. Classification BMI in CRC. b. Classification NRS in CRC. c. Classification PG-SGA in CRC d. Classification Phase angle in CRC. e. Classification VAT in CRC. f. Classification sarcopenia in CRC

Among the patients evaluated, 41 (89.1%) had no oncological treatment prior to surgery and 5 (10.9%) with malignant neoplasia located in the rectum underwent radiotherapy prior to surgery. For patients who had radiotherapy prior to surgery, the genetic study was not performed. The majority of the patients were at nutritional risk (*n* = 29, 64.4%) and moderately malnourished (*n* = 21, 45.7%). However, there was a high percentage of VAT (*n* = 24, 61.5%), increase of VAT/SAT (*n* = 27, 75%), and sarcopenia (*n* = 19, 48.7%) (Table 1). Patients with gene mutations in the tumor had a shorter survival time than patients with the wild type gene, except for *NRAS, BRAF*, and *EGFR* mutations (Table 2). However, these differences were not significant. All nutritional indicators (BMI, nutritional screening, PG-SGA, phase angle, VAT, and sarcopenia) were significantly associated with a higher risk of mortality (all *p* <  0.05, Fig. 2), except VAT/SAT (*p* = 0.366) (Table 3).

**Table 1:** Nutritional status of the patients studied

Nutrition indicators	
BMI (kg/m^2^)	*n* = 46
< 23.0 (Undernourished)^a^ / < 18.5 (Undernourished)^b^	9 (19.6)
23.0–28.0 (normal)^a^ / 18.5–24.9 (normal)^b^	20 (43.5)
28.0–30.0 (Overweight)^a^ / 25–29.9 (Overweight)^b^	6 (13.0)
> 30.0 (obesity)^a^ / > 30.0 (obesity)^b^	11 (23.9)
Nutritional Risk Screening – NRS	*n* = 45
Nutritional risk	29 (64.4)
Without nutritional risk	16 (35.6)
PG – SGA	n = 46
Well nourished	20 (43.4)
Moderetely malnourished	21 (45.7)
Severely malnourished	5 (10.9)
Phase angle	*n* = 41
< 5^o^	15 (36.6)
> 5^o^	26 (63.4)
Visceral adipose tisse	*n* = 39
< 163.8 cm^2^ – male / < 80.1 cm^2^ -female	15 (38.5)
> 163.8 cm^2^ - male / > 80.1 cm^2^ – female	24 (61.5)
VAT /SAT	*n* = 36
< 0.4- male / < 0.4 – female	9 (25)
> 0.4 - male / > 0.4 – female	27 (75)
Sarcopenia	n = 39
Sarcopenic	19 (48.7)
Without sarcopenic	20 (51.3)

*BMI* Body mass index, *PAHO* Pan American Health Organization, *PG-SGA* Patient Self-Produced Global Subjective, *SAT* Subcutaneous adipose tissue, *VAT* Visceral adipose tissue

^a^Classification BMI to elderly -PAHO; ^b^Classification BMI to adults – WHO

**Table 2:** Univariate Kaplan-Meier Survival Analysis according to different clinical characteristics and gene status of oncogenes and tumor suppressor genes from patients with CRC

	*N*	Medium survival (months) (CI 95%)	Log-rank	*p*
Sex				
Male	12	36.4 (26.3–46.6)	0.450	0.502
Female	34	42.1 (35.8–48.4)		
Age				
< 60 years	18	47.4 (40.5–54.3)	3.286	0.070
≥ 60 years	28	33.9 (27.6–40.2)		
Stage I / II / III	28	44.2 (37.8–50.6)	0.669	
IV	18	31.8 (24.0–39.6)		0.413
Tumor site				
Ascending and transverse colon	15	35.9 (27.1–44.7)		
Descending and sigmoid colon	16	42.9 (33.4–52.5)	0.464	0.142
Rectum	15	36.5 (30.0–43.0)		
Gene *PIK3CA* Wild type	14	42.6 (34.4–50.8)	0.936	0.333
Mutated	15	33.9 (25.5–43.4)		
Gene *KRAS*				
Wild type	15	43.3 (35.7–50.9)	1.797	0.180
Mutated	19	33.5 (25.5–41.5)		
Gene *NRAS*				
Wild type	24	38.5 (31.4–45.6)	0.251	0.617
Mutated	5	39.0 (24.6–53.4)		
Gene *BRAF*				
Wild type	24	38.2 (31.0–45.5)	0.426	0.514
Mutated	5	39.8 (26.7–52.8)		
Gene *EGFR*				
Wild type	5	28.8 (22.6–35.1)	0.149	0.700
Mutated	24	38.5 (31.3–45.8)		
Gene *APC*				
Wild type	13	44.5 (36.7–52.3)	1.983	0.159
Mutated	16	32.6 (23.8–41.3)		
Gene *PTEN*				
Wild type	21	38.3 (30.6–46.0)	0.257	0.612
Mutated	8	38.1 (27.2–49.0)		
Gene *SMAD4*				
Wild type	20	40.9 (33.4–48.4)	0.649	0.421
Mutated	9	38.1 (22.2–45.0)		
Gene *FBXW7*				
Wild type	19	39.3 (31.5–47.0)	0.007	0.934
Mutated	10	35.8 (25.0–46.6)		
Total mutations (Oncogenes and Tumor Suppressors)				
< 5 mutations	20	41.0 (33.5–48.4)	0.649	0.421
> 5 mutations	9	33.6 (22.2–45.0)		

*CI* Confidence interval

**Table 3:** Univariate Kaplan-Meier Survival Analysis according different nutritional indicators evaluated in the preoperative period of patients with CRC

Nutrition indicators	N	Medium survival (months) (CI 95%)	Log-rank	P
BMI (kg/m^2^)				
< 23.0 (Undernourished)^a^ / < 18.5 (Undernourished)^b^	9	24.1 (14.0–34.2)	9.297	0.026
23.0–28.0 (normal)^a^ / 18.5–24.9 (normal)^b^	20	35.9 (29.4–42.4)		
28.0–30.0 (Overweight)^a^ / 25–29.9 (Overweight)^b^	6	46.3 (32.2–60.3)		
> 30.0 (obesity)^a^ / > 30.0 (obesity)^b^	11	47.0 (40.1–53.9)		
Nutritional Risk Screening – NRS				
Nutritional risk	29	33.5 (27.4–39.7)	6.405	0.011
Without nutritional risk	16	51.5 (46.4–56.5)		
PG – SGA				
Well nourished	21	47.9 (41.5–54.4)	0.051	0.051
Moderetely malnourished	20	32.6 (25.5–39.6)		
Severely malnourished	5	27.4 (13.1–41.8)		
Phase angle			5.170	0.023
< 5^o^	15	31.8 (23.3–40.4)		
> 5^o^	25	47.5 (41.5–53.4)		
Visceral adipose tisse			4.607	0.032
< 163.8 cm^2^ – male / < 80.1 cm^2^ -female	15	33.0 (22.8–43.3)		
> 163.8 cm^2^ - male / > 80.1 cm^2^ – female	24	44.1 (38.3–50.0)		
VAT /SAT				
< 0.4- male / < 0.4 – female	9	32.7 (22.9–42.4)	0.818	0.366
> 0.4 - male / > 0.4 – female	27	43.1 (36.0–50.2)		
Sarcopenia				
Sarcopenic	19	34.7 (25.2–44.1)	4.930	0.026
Without sarcopenic	20	45.8 (22.9–42.4)		

^*a*^ *Classification BMI to elderly -PAHO;* ^*b*^ *Classification BMI to adults – WHO*

Twelve (26.1%) patients died during the study period. Table 4 presents the crude and age-adjusted hazard ratios (HRs) for death according to different nutritional indicators. A univariate analysis (Crude HR) revealed a higher risk of death among patients with positive indicators for malnutrition or risk of malnutrition when compared to malnourished or overweight patients. Patients at nutritional risk (NRS), moderately/severely malnourished (PG-SGA), and phase angle ≤5 presented a higher risk for death (Table 4).

**Table 4:** Hazard Ratios for death. Brute and adjusted for age. Stage IV of the disease and mutated

Nutrition indicators	HR brute (CI95%)	*p*	HR adjusted (CI 95%) - Age	*p*	HR adjusted (CI 95%) - Stage IV of the disease	*p*	HR adjusted (CI 95%) – Mutated *KRAS* gene	*p*
BMI (kg/m^2^)								
Normal	1.0	–	1.0	–	1.0	–	1.0	–
Undernourished	2.66 (0.88; 8.02)	0.081	2.32 (0.74; 7.23)	0.147	3.02 (0.97; 9.40)	0.056	1.83 (0.49; 6.89)	0.366
Overweight / Obesity	0.31 (0.06; 1.53)	0.153	0.36 (0.07; 1.84)	0.225	0.38 (0.07; 1.93)	0.247	0.30 (0.05; 1.60)	0.160
Nutritional risk screening								
Without nutritional risk	1.0	–	1.0	–	1.0	–	1.0	–
Nutritional rsk	8.77 (1.14; 67.1)	0.036	7.30 (0.88; 59.9)	0.064	8.16 (1.04; 63.7)	0.045	7.24 (0.91; 57.3)	0.060
PG-SGA								
Well nourished	1.0	–	1.0	–	1.0	–	1.0	–
Moderetely / Severely malnourished	3.95 (1.11; 14.0)	0.033	3.10 (0.82; 11.7)	0.095	3.66 (1.02; 13.1)	0.046	4.28 (0.91; 20.1)	0.065
Phase angle								
> 5^o^	1.0	–	1.0	–	1.0	–	1.0	–
< 5^o^	3.79 (1.10; 13.1)	0.035	2.85 (0.73; 11.1)	0.130	7.15 (1.71; 29.8)	0.007	3.07 (0.66; 14.1)	0.151
Visceral adipose tisse								
> 163.8 cm^2^ - male	1.0	–	1.0	–	1.0	–	1.0	–
> 80.1 cm^2^ – female								
< 163.8 cm^2^ – male	3.43 (1.03; 11.4)	0.044	4.08 (1.22; 13.6)	0.022	2.98 (0.88; 10.0)	0.077	4.94 (1.19; 20.5)	0.028
< 80.1 cm^2^ – female								
Sarcopenia								
Without sarcopenic	1.0	–	1.0	–	1.0	–	1.0	–
Sarcopenic	3.95 (1.06; 14.6)	0.040	4.08 (1.22; 13.6)	0.092	3.46 (0.92; 13.0)	0.066	3.12 (0.74; 13.1)	0.121

*HR* Hazard ratio

The HRs and those adjusted for the presence of mutation in the KRAS gene were analyzed according to the different nutritional indicators. A univariate analysis revealed a higher risk of death among patients with positive indicators for malnutrition or risk of malnutrition when compared to patients who were not malnourished or overweight. Patients with VAT < 163.8 cm^2^ (males) or < 80.1 cm^2^ (females) displayed an approximately five-fold increased risk of death when compared to patients with VAT > 163.8 cm^2^ (males) or > 80.1 cm^2^ (females), regardless of the presence of the mutated *KRAS* gene (Table 4).

Cox regression analyses were conducted to investigate the possible predictors of all-cause mortality in the study population. None of the independent prognostic factors had an impact on survival (Table 5).

**Table 5:** Cox regression for all-cause mortality

Nutrition indicators	Exp (B) - 95% CI	*p*-value
BMI	0.656 (0.191–2.250)	0.502
NRS	1.113 (0.132–9.351)	0.922
PG-SGA	0.853 (0.222–3274)	0.816
Phase angle	0.777 (0.178–3.390)	0.737
VAT	0.323 (0.52–1.193)	0.224
Sarcopenia	1.843 (0.360–9.440)	0.463

*BMI* Body mass index, *PG-SGA* Patient Self-Produced Global Subjective, *VAT* Visceral adipose tissue

## Discussion

The study was conducted to verify if the variables involved in nutritional status and genetic mutations correlated with the survival of patients with CRC. Both variables are considered fundamental and determinant in the carcinogenesis and prognosis of these individuals. Obesity is a well-established risk factor for CRC, [4, 5] that influences the treatment and consequent interference with prognosis. [28] In contrast, malnutrition is also associated with therapeutic response and evaluation. [29, 30]

The growing understanding of the involved molecular pathways has revealed that specific gene mutations are determinants in the carcinogenesis and prognosis of CRC. This condition occurs because of loss of gene function due to the mutation influences uncontrolled cell proliferation and apoptosis, and in turn facilitates the development of neoplasms. [3] The loss of function of some CRC-related genes may hinder the therapy with specific biologicals and characterize tumors that are morphologically and histopathologically more difficult to treat, with consequent inference on prognosis. [31, 32]

Advanced age, male gender, advanced stage of disease, and presence of mutations in genes that participate in the carcinogenesis of CRC are variables that influence the shorter survival of patients with CRC. [31, 33] In the present study, clinical and epidemiological characteristics were not associated with mortality risk. However, among the patients with a mutation in the *PIK3CA, KRAS, APC*, or *FBXW7* gene, the survival was lower as well as in individuals who presented with > 5 mutations. The prognostic value of the presence of mutation in genes involved in CRC carcinogenesis is still controversial. Some studies have shown that mutations are associated with poor prognosis, while others report the lack of an association. [33–35]

The lack of an association of the clinical and epidemiological variables to a worsened prognosis can be attributed to the reduced sample size and to the period in which the patients were followed. The median survival of patients with CRC varies with the stage [36, 37].

The nutritional status of cancer patients is a determinant factor in survival, and the presence of nutritional deficits is associated with a worse prognosis. [38] Most of the nutritional indicators that were presently analyzed (BMI, nutritional screening, PG-SGA, phase angle, VAT, and sarcopenia) were associated with a higher risk of mortality. Regardless of the most advanced stage of the disease (stage IV), nutritional deficiency evident as low weight, nutritional risk, moderately/severely malnourished, and phase angle <5^o^ was related to the higher risk of death.

In this study, individuals at nutritional risk or moderately/severely malnourished had decreased survival. Screening and early nutritional assessment can identify the risk of malnutrition, minimize weight loss, and indicate patients who will benefit from early and specialized nutritional intervention. [39] In addition to this application, these tools are also useful for predicting the morbidity of cancer patients. [40, 41]

Increasing interest in the potential of the phase angle to predict adverse outcomes, such as mortality, may reflect cell size, cell membrane integrity and/or water distribution in the extra and intracellular compartments. [41, 42] Many studies have reported that a low phase angle is associated with decreased survival in cancer patients. [41–43] Hui et al. [43] evaluated patients hospitalized with advanced cancer and determined that the median survival was 106 days and that the reduced phase angle was associated with worse survival independent of other known prognosis, such as palliative prognostic score, palliative prognostic index, lean mass and hypoalbuminemia. Another study also identified an average survival time of 250 days when the mean phase angle was 4.4 (±1.0) in outpatient palliative care patients. [44] In the present study, patients with a phase angle <5^o^ were associated with lower survival.

Patients with sarcopenia tend to have lower survival compared to patients without sarcopenia, [45] as also observed in the present study. With regard to postoperative mortality, Boer et al. [46] found in a series of 91 patients who underwent surgery for CRC resection that sarcopenia was a predictor for worse survival, reducing sarcopenia by 1 year. Similarly, Reisinger et al. [47] reported that sarcopenia was a predictor for worse survival within 30 days. In our analysis, of the 11 patients who died, 7 had sarcopenia. However, because of the small sample size, it was not possible to associate sarcopenia with mortality. The present study classified sarcopenia by means of the height-related muscle mass index, as proposed by some authors. [48, 49] On the contrary, it is known that sarcopenia should be understood by the association of muscle mass reduction plus the interpretation of functional capacity changes, a variable that was not analyzed in the study.

A recent cohort study identified for the first time that low muscle mass or sarcopenia is also highly prevalent among patients with non-metastatic CRC and that the adverse effect is not restricted to patients with advanced CRC associated with cachexia. The study cited similar results to the present study, in which almost 45% of newly diagnosed men and 40% of women had sarcopenia. These patients compared to those without sarcopenia had a 30% overall mortality risk and a 50% increased risk of CRC without metastasis. [50] In the present study, it was observed that the frequency of sarcopenia was also present in individuals at the initial stage of the disease.

Excess weight translated by BMI and VAT seems to exert a beneficial effect on survival. Some evidence supports the view that high BMI interferes with the worse prognosis of patients with CRC. However, several recent studies involving cancer patients reported that elevated BMI was associated with improved survival compared to patients with normal weight. [10, 11] BMI is a measure that is readily available in patients with CRC. However, BMI does not accurately measure adiposity or muscle mass. [12] The few studies that have been able to directly measure body composition have shown that low muscle mass [48, 51] or greater visceral adiposity [52, 53] are associated with worse survival. However, most of these studies have been very small (< 250 patients) and were performed in patients with advanced cancer with poor prognosis.

A recent retrospective cohort study concluded that lower adiposity is an independent factor associated with increased risk of mortality after adjustment for the major predictors of mortality in cancer patients. [54] Among the deposits of adipose tissue, SAT maintained the prognostic value on the VAT. When the combination of adiposity and sarcopenia was considered, the presence of sarcopenia and low SAT were associated with lower survival. However, the effect of sarcopenia on survival was more pronounced in patients with low subcutaneous adiposity. Therefore, in the absence of sarcopenia, high adiposity has been shown to be a phenomenon of protective body composition and is advantageously associated with the survival of oncological patients. [54] These results corroborate the findings of the present study, since of the 24 patients with increased VAT, only five had sarcopenia.

To understand the role that body composition can play in cancer patients in relation to prognosis, the profile of the patients who received interventions with the purpose of promoting muscular anabolism and maintenance of SAT may be instructive to examine.

Even with the sample size limitation, the “protective” role of increased VAT for survival remained in those older than 60 years and in the presence of the mutated *KRAS*. Both variables directly influence the lower survival. When adjusting the risk of mortality with the advanced stage of the disease, the results were marginally significant. Use of larger number of patients might reveal a relationship between the variables.

This study has some limitations. Paramount was the sample size, since the reduced number of patients included may reduce the statistical power of some analyses. Another limitation was the prevalence of women in the study group. This condition could influence the interpretation of data related to body composition. To control this condition, classifications of variables related to composition were stratified according to gender. A study strength was its prospective design, in contrast to previous studies.

## Conclusion

In conclusion, it was possible to observe a higher risk of death among patients with positive indicators for malnutrition or risk of malnutrition, according to BMI, NRS, PG-SGA, phase angle, VAT, and sarcopenia, when compared to non-malnourished or overweight patients. No relationship was identified between the presence of mutations and survival. Considering that variables involved in nutritional status and genetic mutations are fundamental and are important in carcinogenesis and prognosis of cancer patients, more research is needed to elucidate the impact of their associations on survival. With this understanding, it will be possible to propose more effective clinical and nutritional therapeutic interventions.

## Data Availability

The data that support the findings of this study are available from the corresponding author but restrictions apply to the availability of these data, which were collected for the current study, and so are not publicly available. Data are however available from the authors upon reasonable request.

## Abbreviations

BIA: Bioelectrical impedance analysis
BMI: Body mass index
CIN: Chromosomal instability
CRC: Colorectal cancer
CT: Computed tomography
L3: third lumbar vertebra
PA: Phase angle
PG-SGA: Patient Self-Produced Global Subjective
R: Resistance
SAT: Subcutaneous adipose tissue
SMT: Skeletal muscle tissue
VAT: Visceral adipose tissue
Xc: Reactance
